# News and Notes

**Published:** 1913-04

**Authors:** 


### NEWS AND NOTES

### PRELIMINARY PROGRAM, AMERICAN PROCTOLOGIC SOCIETY.—FIFTEENTH ANNUAL MEETING

MINNEAPOLIS, MINN., JUNE 16 AND 17, 1913

---

*Headquarters and Place of Meeting, Hotel Radisson, Seventh Street, near Nicolet Avenue. The Profession is Cordially Invited to Attend all Meetings*

PROGRAM

Commencing Monday, June 16, 1913:

Executive Council meets at 11 A. M.

First regular session at 2 P. M.

Annual address of the President—Subject: Proctology and Procto-Enterology, Louis J. Hirschman, Detroit, Mich.

Memoir of James P. Tuttle, New York City, N. Y. Joseph M. Mathews, Louisville, Ky.

Memoir of Leon Straus, St. Louis, Mo., Joseph M. Mathews, Louisville, Ky

PAPERS

1—A Review of Proctologic Literature for 1912: Samuel T. Earle, Baltimore, Md.

2—A Method of Operating on Fistula Without Cutting Muscular Tissue: Rollin H. Barnes, St. Louis, Mo.

3—Report of a Case of Fecal Tumor Associated with Hirschsprung's Disease: Alois B. Graham, Indianapolis, Ind.

4—A Further Consideration of Sir Charles Ball's Operation on Internal Hemorrhoids: Alfred J. Zobel, San Francisco, Cal.

5—Deductions Based Upon an Analysis of Four Thousand Consecutive Rectal Cases: T. Chittenden Hill, Boston, Mass.

6—Personal Reminiscences Upon the Subject of Proctology: Jos. M. Mathews, Louisville, Ky.

7—Plastic Operations in Anal Stricture: Wm. M. Beach, Pittsburgh, Pa.

8—Injection Treatment of Hemorrhoids: Lewis H. Adler, Jr., Philadelphia, Pa.

9—Anal Sphincters: Ralph W. Jackson, Fall River, Mass.

10—Further Observations Upon the Surgical Anatomy and Pathology of the Large Bowel with Radiographic Illustrations: Granville S. Hanes, Lousville, Ky.

11—The Ano-rectal Line: Its Clinical Significance: Collier F. Martin, Philadelphia, Pa.

12—Intestinal Parasitism in the South: Modes of Distribution: A National Problem: John L. Jelks, Memphis, Tenn.

13—Some Preliminary Observations on Gastro-Enteric Motility: Jerome M. Lynch, New York City, N. Y.

14—Ano-rectal Fibrosis: A New Disease: J. Coles Brick, Philadelphia, Pa.

15—Some New Diagnostic Means of Investigating Diseases of the Gastro-Intestinal Tact: Thos. Chas. Martin, Washington, D. C.

16—Carcinoma of the Rectum: J. Rawson Pennington, Chicago, Ill.

17—Venereal Affections of the Anus and Rectum: Edw. A. Hamilton, Columbus, Ohio.

18—Further Observations on the Treatment of Pruritis Ani by Autogenous Vaccines: Dwight H. Murray, Syracuse, N. Y.

19—Diarrhea: Its Causes and Treatment: George B. Evans, Dayton, Ohio.

20—Ulcerations of the Rectum and Their Treatment: Horace Heath, Denver, Col.

---

Candidates for Active or Associate Fellowship 1913

1. William H. Axtell, Exchange Block, Bellingham, Wash.
2. Lee J. Erastberger, 711 State Natl. Bldg. Oklahoma City, Okla.
3. V. Lee Fitzgerald, 17 Batley St., Providence, R. I.
4. J. M. Frankenburger, Rialto Bldg., Kansas City, Mo.
5. Wm. H. Kiger, 308 Consolidated Bank Bldg., Los Angeles, Cal.

6. Walter Irwin Le Fevre, 218 Lennox Bldg., Cleveland, Ohio.

7. Treby W. Lyon, 315 W. 51st St., New York City.

Associate Fellows

Rollin H. Barnes, Metropolitan Bldg., St. Louis, Mo.
Barney J. Dryfuss, 62 W. 89th St., New York City, N.Y.
James A. Duncan, 1107 Broadway, Toledo, Ohio.
Arthur E. Holding, 103 Park Ave., New York City, N. Y.
Ralph W. Jackson, 245 Cherry St., Fall River, Mass.
E. H. Terrell, 304 E. Grace St., Richmond, Va.

